# Effects of garlic *(Allium sativum L)* and *Citrullus colocynthis* (L.) Schrad individually and in combination on male reproductive damage due to diabetes: suppression of the AGEs/RAGE/Nox-4 signaling pathway

**DOI:** 10.1186/s12906-024-04402-8

**Published:** 2024-04-05

**Authors:** Aghileh Mohammadzadeh, Ali Gol, Reza Kheirandish

**Affiliations:** 1 Department of Biology, Faculty of Science, Shahid Bahonar University of Kerman, Kerman, Iran; 2 Department of Pathobiology, Veterinary Faculty, Shahid Bahonar University of Kerman, Kerman, Iran

**Keywords:** Diabetes mellitus, Garlic, *Citrullus colocynthis*, Oxidative stress, Apoptosis

## Abstract

**Background:**

Diabetes Mellitus is associated with disturbances in male reproductive function and fertility. Studies have shown that oxidative stress with the subsequent inflammation and apoptosis cause these complications in diabetes. Garlic (G) (*Allium sativum L*) and *Citrullus colocynthis (L.) Schrad* (C) both have antidiabetic and antioxidant properties. Recently, we demonstrated their synergistic effects in alleviating reproductive complications when administered concomitantly. However, as even medicinal plants in long term usage may lead to some unwanted side effects of their own, we examined whether with half the original doses of these two medicinal plants we could achieve the desired results.

**Methods:**

Thirty-five male Wistar rats were divided into five groups (*n* = 7/group): Control, Diabetic, Diabetic + G (0.5 ml/100 g BW), Diabetic + C (5 mg/kg BW) and Diabetic + GC (0.5 ml/100 g BW of garlic and 5 mg/kg BW of *C. colocynthis*) groups. The experimental period was 30 days.

**Results:**

Oxidative stress, advanced glycation end products (AGEs), immunoexpression of caspase-3, and expression of mRNAs for receptor for advanced glycation end products (RAGE), NADPH oxidase-4 (NOX-4) and nuclear factor kappa B increased in testis of diabetic rats. Treatment with garlic and *C. colocynthis* alone showed some beneficial effects, but in the combination form the effectiveness was more profound.

**Conclusions:**

We conclude that the combination therapy of diabetic rats with lower doses is still as efficient as higher doses; therefore, the way forward for reducing complications in long term consumption.

## Background

Diabetes mellitus (DM) is a chronic metabolic disease that has become a growing global problem [1]. International Diabetes Federation states that the number diabetic patients worldwide is projected to increase to 629 million by 2045 [2]. DM negatively affects reproductive function, especially in men at the peak of their reproductive years, thus contributing to a general reduction in fertility rate [3]. Testicular oxidative stress, inflammation, and apoptosis, observed in diabetes mellitus, is associated with reduced fertility [4–6].

Advanced glycation end products (AGEs) play a causative role in the development and progression of DM complications [7]. AGEs belong to a heterogeneous group of compounds formed from non-enzymatic glycation of proteins, and their production is accelerated as a result of hyperglycemia [8]. Receptors for advanced glycation end products (RAGE) mediates AGEs effects [9].

The interaction between AGEs and RAGE leads to NADPH oxidase (Nox) activation, Reactive Oxygen Species (ROS) production, activation of NF-кB-mediated genes transactivation, and increases in inflammatory cytokines accumulation [9]. Thus, inhibition of the downstream signaling pathway induced by the AGEs-RAGE interaction may be a strategy to prevent DM-induced male reproductive injury.

Current synthetic medicines lack to reverse hyperglycemia completely, possess limited tolerability as well as side effects. Therefore, there is a need for alternative therapies, especially plant sources with maximal efficacy and minimal side effects [10]. Even WHO Committee has emphasized that their effectiveness in managing DM should be examined [11]. In some traditional medicines, DM is better managed by combining plants instead of single plants, due to synergy and fewer side effects. There are about two hundred herbal formulations on the markets for DM, most of which contain eight or more plants in combination that are very complex to standardize; therefore, product quality cannot be guaranteed. The FDA recommends that the number of ingredients in each compound be three or less [12].

*Citrullus colocynthis* (L.) Schrad (*C. colocynthis*) is a member of the Cucurbitaceae family [13]. It is a perennial herbaceous vine that produces 15 to 30 round fruits 7–10 cm in diameter [14]. It is widely distributed in desert areas especially in the Sahara Desert and Southwest Asia, including the deserts of Saudi Arabia, Pakistan, India, China, and Iran. Several bioactive compounds have been reported for *C. colocynthis* fruit, including flavonoids such as catechin and quercetin [15]. In recent decades, numerous studies have demonstrated remarkable biological functions for *C. colocynthis*, including antioxidative [16], anti-cancer [17], anti-inflammatory, antipyretic, analgesic [18], anti-bacterial [19], and anti-diabetic [14]. Also, the effectiveness of *C. colocynthis* on complications caused by DM has been investigated. One of these complications is reproductive complications, with few studies in this field.

Garlic (*Allium sativum* L.) is a perennial herbaceous plant and has been used as a traditional medicine since ancient times. It is believed to be native to Central Asia and Northeastern Iran [20]. Garlic possesses antioxidant, anti-cancer, antifungal, hepatoprotective, anti-inflammatory, and antidiabetic properties. It has various bioactive compounds such as organic sulfur compounds, saponins, phenolic compounds and polysaccharides. The main active components of this plant are organosulfur compounds, including diallyl thiosulfinate (allicin), diallyl sulfide (DAS), diallyl disulfide (DADS), diallyl trisulfide (DATS), S-allyl cysteine (SAC), and S-allyl-cysteine sulfoxide (alliin) [21]. Studies have shown the beneficial effects of garlic on complications caused by DM, including damage to the brain [22], retina [23], kidney [24] and heart [25]. However, the effect of garlic on testicular injury caused by DM has been less investigated.

Despite their beneficial effects in managing many disorders, medicinal plants are able to induce complications in long term usage [26]. Therefore, the dosage needs to be taken into account to have both the more effectiveness and less side effects.

Recently, we have shown the synergistic effects of garlic and *C. colocynthis* consumption in alleviating testicular injuries [27].

In this study, we aimed to assess the protective effects of garlic and *C. colocynthis* administration to find their desired results even in half the doses in our other study.

This study evaluates the therapeutic effect of garlic and *C. colocynthis,* individually and in combination, on male reproductive injury caused by DM. The AGEs/RAGE/Nox-4 signaling pathway, which causes oxidative stress during the pathological process, also will be discussed.

## Materials and methods

### Collection and preparation of plant material

*C. colocynthis* fruit was collected from Sarzeh village, Sistan and Baluchestan province of Iran and its pulp was ground to prepare powder.

White garlic was purchased from the local market and was used to make aqueous extract based on the method described by Masjedi et al. [28].

Both plants were identified by Dr. SM. Mirtadzaddini from the Department of Biology, Shahid Bahonar University of Kerman, Iran (3836 MIR for *Citrullus colocynthis* and 3836 MIR for garlic).

### Animals

Male Wistar rats (weighing 220–250 g, 9 weeks old) from the Animal House unit of Shahid Bahonar University of Kerman, Iran were adapted to standard laboratory conditions (temperature 23 ± 2 °C with alternating 12 h light/dark cycles) for one week before the experiments. Rats were allowed ad libitum access to normal pellet and water diet.

### Induction of diabetes in experimental animals

Rats were fasted overnight and then STZ (60 mg/kg) was injected intraperitoneally. Fasting blood glucose (FBG) was assessed using a glucometer (ARKRAY Glucocard 01, Japan) 72 h after STZ injection and rats with blood glucose levels above 250 mg/dL were considered diabetic and used for further experiments.

### Experimental design

Thirty-five rats were randomly divided into 5 groups (*n* = 7) as follows: (I) Control group, (II) Diabetic group, (III) diabetic group receiving 0.5 ml/100 g BW of aqueous extract of garlic (Diabetic + G), (IV) diabetic group receiving 5 mg/kg BW of *C. colocynthi*s pulp powder (Diabetic + C), and (V) diabetic group receiving 0.5 ml/100 g BW of aqueous extract of garlic and 5 mg/kg BW of *C. colocynthi*s pulp powder (Diabetic + GC).

At the end of the experiment, rats were kept fasted overnight and the following morning they were anesthetized with CO_2_ and the chest cavity was opened and blood was collected through cardiac puncture. Then, the rats were killed by cutting the heart. In the end, by an incision in the abdomen, the testes and cauda epididymis were isolated and washed in cold normal saline and weighed. The left testis was kept at -80°C for oxidative stress analysis and gene expression and the right testis was kept at 10% formalin for histological examination. Cauda epididymis was used immediately for sperm count.

### Serum preparation and hormonal tests

Blood samples were coagulated at room temperature and centrifuged at 3,500 rpm for 15 min﻿ and the serum was separated and stored at -20°C for hormonal analysis. Serum LH and testosterone levels were determined by enzyme-linked immunosorbent assay (ELISA) (ELISA Microplate Reader, Vira)﻿.

### Sperm count

The right cauda epididymis was weighed and then minced in phosphate-buffered saline (PBS) at 35–37° C. After incubation at 37° C, 0.5 ml of the sperm suspension was transferred to a tube containing 2 ml of PBS. In the end, 10 μl of the sample was loaded on one side of the hemocytometer, and the sperms were counted in five secondary squares [29].

### AGEs assay

AGEs were determined by spectrofluorometer (Cary Eclipse Fluorescence Spectrometer, Agilent, USA) based on their fluorescence intensity scan. Testis homogenates were diluted with phosphate buffer solution (pH 7.4), and fluorescence intensity was recorded at excitation of 350 nm and emission at 440 nm [30].

### RNA extraction, cDNA synthesis, and real-time PCR

Total RNA was extracted from testis using the trizol reagent. Primers for the genes of NF-кB, RAGE, Nox-4 and GAPDH (housekeeping gene) were synthesized by Gene Fanavaran Ltd Company, Iran and primer sequences are shown in Table 1. The expression of GAPDH was used as a standard control. After RNA extraction, cDNA samples were made using Parstous kit (Easy cDNA Synthetic Kit (A101161). RT-qPCR was performed using Rotor-Gene. To calculate the relative levels of mRNA, method ΔCT was used.

**Table 1:** Primers used for RT-qPCR analysis

Gene size	Primer sequence	Accession	Amplicon
		Number	
GAPDH	F: CAAGCCTGAGAATGGGAAGC R: GAAGACGCCAGTAGACTCCA	NM_017008.4	127
RAGE	F: AACCCAGACTCGAGGAGAGGAA R: TGAGGTCGGAAGCTGAAGGA	XM_006256025.3	158
Nox-4	F: CTTTTTATTGGGCGTCCTC R: GGTCCACAGCAGAAAACTCC	XM_008759643.3	92
NF-кB	F: AGAGCAACCGAAACAGAGAGG R: ATATGCCGTCCTCACAGTGC	NM_001276711.1	227

*GAPDH* Glyceraldehyde-3-phosphate dehydrogenase, *RAGE* Receptor for advanced glycation end products, *Nox-4* NADPH oxidase-4, *NF-кB* Nuclear factor kappa B

### Oxidative stress of testis

The supernatants from the testis was used for analyzing malondialdehyde (MDA) [31], catalase (CAT) [32], glutathione peroxidase (GPx) [33], glutathione (GSH) and GSSG [32].

### Histopathological examinations of the testis

The right testis was fixed in 10% formalin for 72 h. The fixed testis was dehydrated using increasing concentrations of alcohol. Before embedding in paraffin, it was cleared by xylene. Paraffinized tissues were molded and cut into 5 μm thick sections using a microtome, and then fixed on slides. Thereafter, paraffin was removed and hydration was done by decreasing concentrations of alcohol, and the tissues were stained with hematoxylin and eosin. Sections of testicular tissue were observed under a light microscope (Nikon, 50i, USA) and then photographed (Nikon, DS-Fi2, USA).

### Immunohistochemical examination of the testis

Sections with 5 μm thickness were used for immunohistochemistry. Slides were placed in the 1X tris buffered saline solution in the microwave and it was turned off after reaching the boiling point, leaving the samples in the solution for 20 min. Samples were washed with PBS. In order to block cellular peroxidase, H_2_O_2_ and methanol were mixed with a ratio of 1 to 9 and placed on the samples for 10 min. Thereafter, the primary antibody diluted with PBS was poured on them and they were placed at room temperature for one hour. After that, an appropriate amount of diluted secondary antibody was poured on each slide. Next, an appropriate amount of diluted Sav-HRP was poured on the samples and kept in the dark for 30 min. Afterward, the DAB solution was added to the samples. The samples were washed with water after 5 min and finally immersed in hematoxylin dye for 10 s.

### Statistical analysis

Data are analyzed by one-way analysis of variance followed by post-hoc Tukey test by using IBM SPSS (version 20, Armonk, New York, USA) package. Results are presented as mean ± SEM. Level of significance was at *p* < 0.05.

## Results

### Fasting blood glucose, and body and testis weight

FBG was significantly increased in Diabetic (*p* < 0.001), Diabetic + G and Diabetic + C groups (*p* < 0.01) compared to the Control group. In Diabetic + GC group, it decreased significantly compared to Diabetic (*p* < 0.001) and Diabetic + G (*p* < 0.05) groups (Table 2). Body weight in Diabetic, Diabetic + G and Diabetic + C groups (*p* < 0.001), and Diabetic + GC group (*p* < 0.05) showed a significant decrease compared to control group. In Diabetic + GC group it had a significant increase (*p* < 0.01) compared to the Diabetic and Diabetic + C groups (Table 2). Table 2 shows that testis weight decreased in Diabetic (*p* < 0.001), Diabetic + G (p < 0.05) and Diabetic + C (*p* < 0.05) groups compared to the Control group. It had a significant increase in Diabetic + GC group compared to Diabetic (*p* < 0.01), Diabetic + G (*p* < 0.05) and Diabetic + C (*p* < 0.05) groups.

**Table 2:** The effect of garlic and *C. colocynthis* on FBG and body and testis weight

**Groups**	FBG (mg/dl)	Body weight (g)	Testis weight (g)
Control	59.5 ± 4	238.55 ± 4.15	1.21 ± 0.033
Diabetic	337 ± 36.9^***^	164.8 ± 5.08^***^	0.89 ± 0.0305^***^
Diabetic + G	260.66 ± 35.7^**^	179.42 ± 9.7^***^	0.996 ± 0.0598^*^
Diabetic + C	244 ± 25.12^**^	168.36 ± 9.37^***^	0.973 ± 0.0634^*^
Diabetic + GC	119.83 ± 27.6^###,$^	205.72 ± 4.33^*,##,&&^	1.206 ± 0.0438^##,$,&^

Diabetic + G: Diabetic rats treated with garlic at a dose of 0.5 ml/100g BW. Diabetic + C: Diabetic rats treated with *C. colocynthis* at a dose of 5 mg/kg BW. Diabetic + GC: Diabetic rats treated with garlic and *C. colocynthis* at a dose of 0.5 ml/100g BW and 5 mg/kg BW respectively

Values are mean ± SEM (*n* = 7)

^***^p < 0.001 against Control group

^**^p < 0.01 against Control group

^*^p < 0.05 against Control group

^###^*p* < 0.001 against Diabetic group

^##^*p* < 0.01 against Diabetic group

^$^*p* < 0.05 against Diabetic + G group

^&&^*p* < 0.01 against Diabetic + C group

^&^*p* < 0.05 against Diabetic + C group

### LH and Testosterone levels and sperm count

**Fig. 1:**
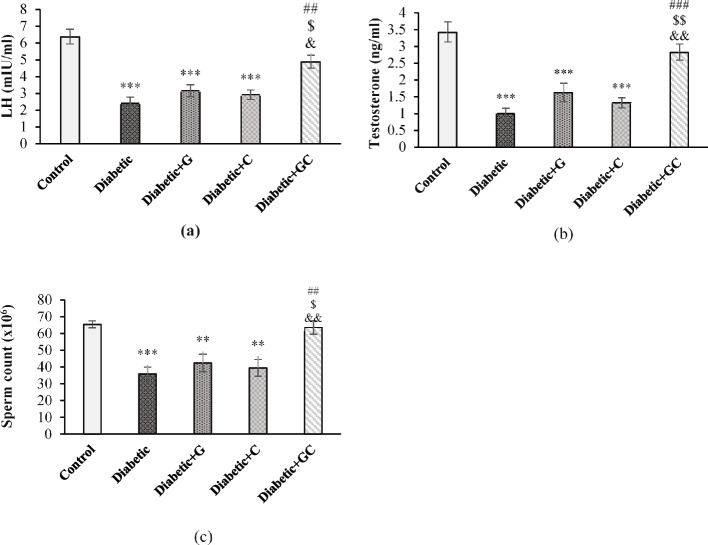
The effect of garlic and *C. colocynthis* on LH (**a**) and testosterone levels (**b**) and sperm count (**c**). Values are mean ± SEM (*n* = 7). *** *p* < 0.001 against Control group. ** *p* < 0.01 against Control group. ### *p* < 0.001 against Diabetic group. ## *p* < 0.01 against Diabetic group. $$ *p* < 0.01 against Diabetic + G group. $ *p* < 0.05 against Diabetic + G group. && *p* < 0.01 against Diabetic + C group. & *p* < 0.05 against Diabetic + C group. Diabetic + G: Diabetic rats treated with garlic at a dose of 0.5 ml/100g BW. Diabetic + C: Diabetic rats treated with *C. colocynthis* at a dose of 5 mg/kg BW. Diabetic + GC: Diabetic rats treated with garlic and *C. colocynthis* at a dose of 0.5 ml/100g BW and 5 mg/kg BW respectively

LH was significantly decreased in Diabetic, Diabetic + G and Diabetic + C groups (*p* < 0.001) compared to the Control group. It increased in Diabetic + GC group significantly compared to the Diabetic (*p* < 0.001), and Diabetic + G, and Diabetic + C groups (*p* < 0.05) (Fig. 1a). Figure 1b shows that testosterone level reduced in Diabetic, Diabetic + G and Diabetic + C groups (*p* < 0.001) compared to Control group, but in Diabetic + GC group it increased significantly compared to Diabetic (*p* < 0.001), and Diabetic + G and Diabetic + C groups (*p* < 0.01). Sperm count in Diabetic group (*p* < 0.001), and Diabetic + G and Diabetic + C groups (*p* < 0.01) decreased significantly compared to the Control group. In Diabetic + GC group it increased significantly compared to Diabetic (*p* < 0.01), Diabetic + G (*p* < 0.05), and Diabetic + C (*p* < 0.01) groups (Fig. 1c).

### AGEs level

**Fig. 2:**
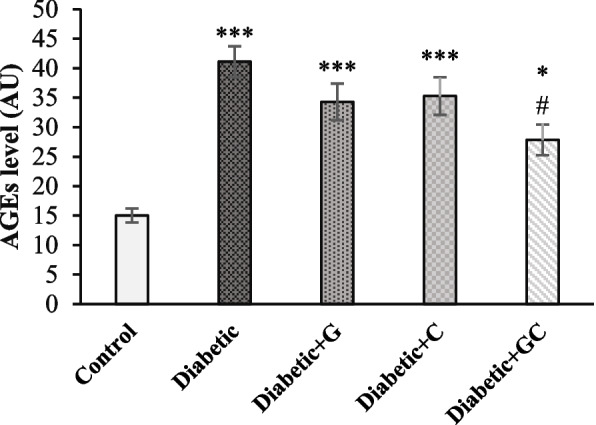
The effect of garlic and *C. colocynthis* separately and in combination on AGEs level. Values are mean ± SEM (*n* = 7). *** *p* < 0.001 against Control group. * *p* < 0.05 against Control group. # *p* < 0.05 against Diabetic group. Diabetic + G: Diabetic rats treated with garlic at a dose of 0.5 ml/100g BW. Diabetic + C: Diabetic rats treated with *C. colocynthis* at a dose of 5 mg/kg BW. Diabetic + GC: Diabetic rats treated with garlic and *C. colocynthis* at a dose of 0.5 ml/100g BW and 5 mg/kg BW respectively

AGEs of Diabetic group, Diabetic + G & Diabetic + C groups (*p* < 0.001), and Diabetic + GC group (*p* < 0.05) showed a significant increase compared to Control group. However, its level in Diabetic + GC group was significantly lower than the Diabetic group (*p* < 0.05) (Fig. 2).

### mRNA expression levels of RAGE, Nox-4 and NF-кB

**Fig. 3:**
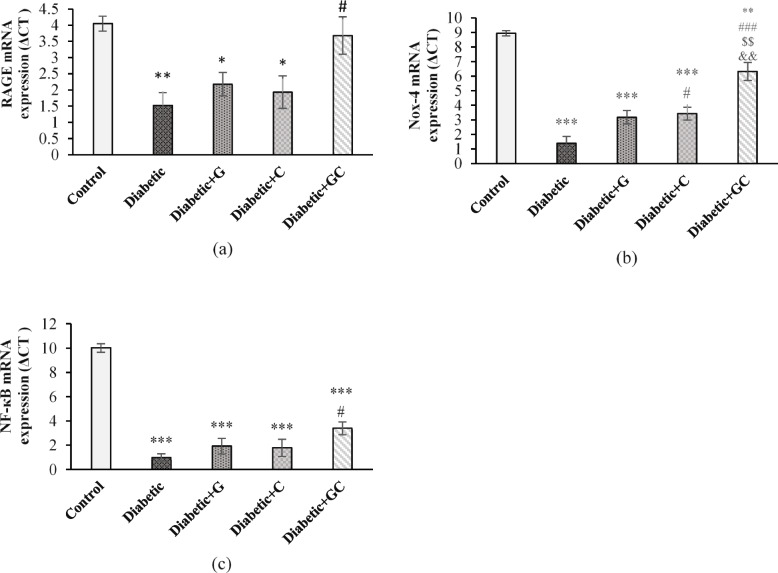
The effect of garlic and *C. colocynthis* and their combination on mRNA expression of RAGE (**a**), Nox-4 (**b**), and NF-кB (**c**). Values are mean ± SEM (*n* = 5). *** *p* < 0.001 against Control group. ** *p* < 0.01 against Control group. * *p* < 0.05 against Control group. # *p* < 0.05 against Diabetic group. & *p* < 0.05 against Diabetic + C group. Diabetic + G: Diabetic rats treated with garlic at a dose of 0.5 ml/100g BW. Diabetic + C: Diabetic rats treated with *C. colocynthis* at a dose of 5 mg/kg BW. Diabetic + GC: Diabetic rats treated with garlic and *C. colocynthis* at a dose of 0.5 ml/100g BW and 5 mg/kg BW respectively

Expression of RAGE was significantly increased in Diabetic (*p* < 0.01), Diabetic + G (*p* < 0.05), and Diabetic + C (*p* < 0.05) groups compared to the Control group. However, its expression in the Diabetic + GC group had a significant decrease (*p* < 0.05) compared to the Diabetic group (Fig. 3a).

Nox-4 expression was significantly increased in Diabetic, Diabetic + G and Diabetic + C groups (*p* < 0.001), and Diabetic + GC group (*p* < 0.05) compared to the Control group. It decreased in Diabetic + GC (*p* < 0.05) group compared to Diabetic group. Also, its expression in Diabetic + GC group (*p* < 0.05) was significantly reduced compared to Diabetic + G and Diabetic + C groups (Fig. 3b).

NF-κB expression was significantly increased in Diabetic, Diabetic + G and Diabetic + C groups (*p* < 0.001) compared to the Control group. In Diabetic + GC group it decreased (*p* < 0.05) compared to Diabetic group (Fig. 3c).

### Oxidative stress markers

**Fig. 4:**
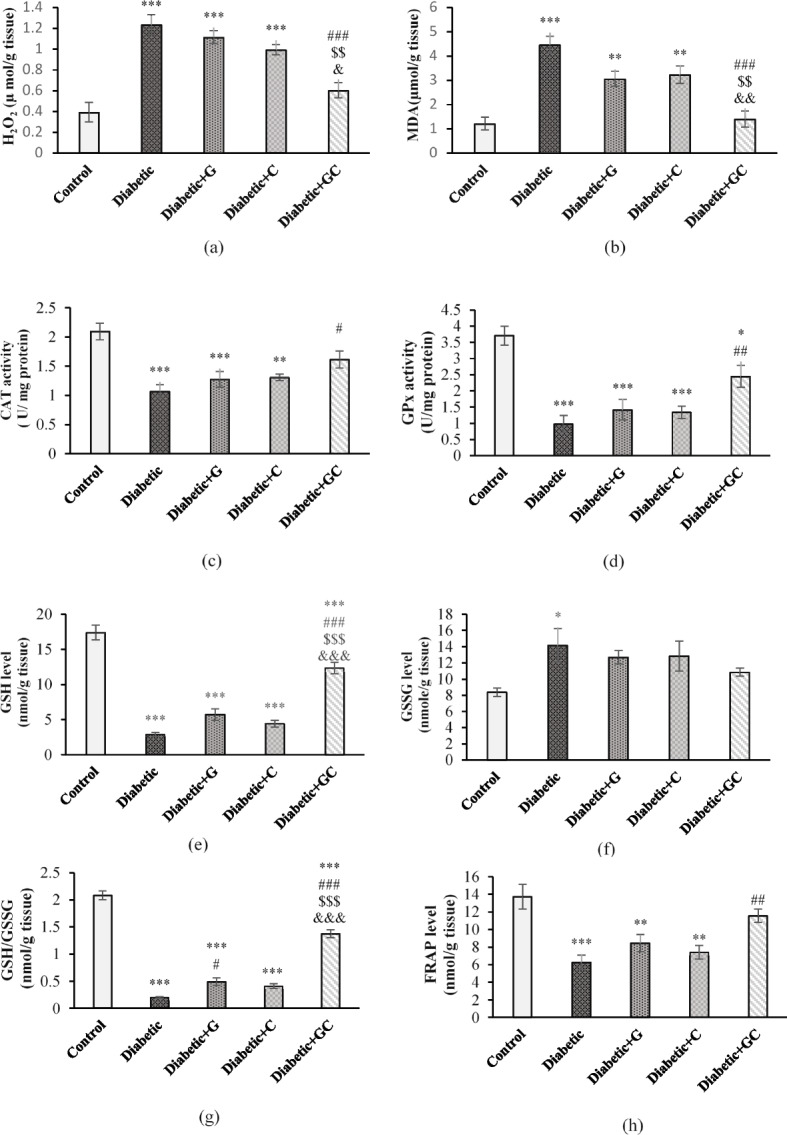
The effect of garlic and *C. colocynthis* and their combination on H_2_O_2_ (**a**) and MDA levels (**b**), CAT (**c**) and GPx activity (d), GSH (**e**) and GSSG (**f**) levels, GSH/GSSG ratio (**g**) and FRAP level (**h**). Values are mean ± SEM (*n* = 7). *** *p* < 0.001 against Control group. ** *p* < 0.01 against Control group. * *p* < 0.05 against Control group. ### *p* < 0.001 against Diabetic group. ## *p* < 0.01 against Diabetic group. # *p* < 0.05 against Diabetic group. $$$ *p* < 0.001 against Diabetic + G group. $$ *p* < 0.01 against Diabetic + G group. &&& *p* < 0.001 against Diabetic + C group. && *p* < 0.01 against Diabetic + C group. & *p* < 0.05 against Diabetic + C group. Diabetic + G: Diabetic rats treated with garlic at a dose of 0.5 ml/100g BW. Diabetic + C: Diabetic rats treated with *C. colocynthis* at a dose of 5 mg/kg BW. Diabetic + GC: Diabetic rats treated with garlic and *C. colocynthis* at a dose of 0.5 ml/100g BW and 5 mg/kg BW respectively

H_2_O_2_ level of Diabetic, Diabetic + G & Diabetic + C groups showed a significant increase compared to Control group (*p* < 0.001). However, its level in Diabetic + GC group was significantly lower than the Diabetic group (*p* < 0.001) and Diabetic + G (*p* < 0.01) & Diabetic + C (*p* < 0.05) groups (Fig. 4a).

MDA level of Diabetic group and Diabetic + G & Diabetic + C groups showed a significant increase compared to Control group (*p* < 0.001 & *p* < 0.01). However, its level in Diabetic + GC group was significantly lower than the Diabetic group (*p* < 0.001) and Diabetic + G & Diabetic + C groups (*p* < 0.01) (Fig. 4b).

CAT activity showed a significant decrease in Diabetic (*p* < 0.001), Diabetic + G (*p* < 0.001) & Diabetic + C (*p* < 0.01) groups compared to Control group. However, its activity in Diabetic + GC group showed an increase compared to Diabetic group (*p* < 0.05) (Fig. 4c).

GPx activity was significantly reduced in Diabetic, Diabetic + G, and Diabetic + C groups (*p* < 0.001) and Diabetic + GC group (*p* < 0.05) compared to Control group. However, its activity was significantly restored in the Diabetic + GC group compared to Diabetic group (*p* < 0.01) (Fig. 4d).

GSH level in Diabetic, Diabetic + G, Diabetic + C and Diabetic + GC groups exhibited a significant decrease compared to the Control group (*p* < 0.001). Its level in Diabetic + GC group increased significantly compared to Diabetic, Diabetic + G and Diabetic + C groups (*p* < 0.001) (Fig. 4e).

Level of GSSG in the Diabetic group was significantly increased relative to the Control group (*p* < 0.05) and there was no significant difference between all treatment groups with Control group (Fig. 4f).

GSH/GSSG ratio was significantly lower in all Diabetic groups than in the Control group (*p* < 0.001). This ratio in Diabetic + GC group showed a significant increase compared to the Diabetic, Diabetic + G, and Diabetic + C groups (*p* < 0.001). Also, it increased significantly in Diabetic + G group compared to Diabetic group (*p* < 0.05) (Fig. 4g).

FRAP level of Diabetic group and Diabetic + G & Diabetic + C groups showed a significant increase compared to Control group (*p* < 0.001 & *p* < 0.01). However, its level in Diabetic + GC group was significantly lower than the Diabetic group (*p* < 0.01) and Diabetic + G & Diabetic + C groups (*p* < 0.05) (Fig. 4h).

### Histopathology of testis

**Fig. 5:**
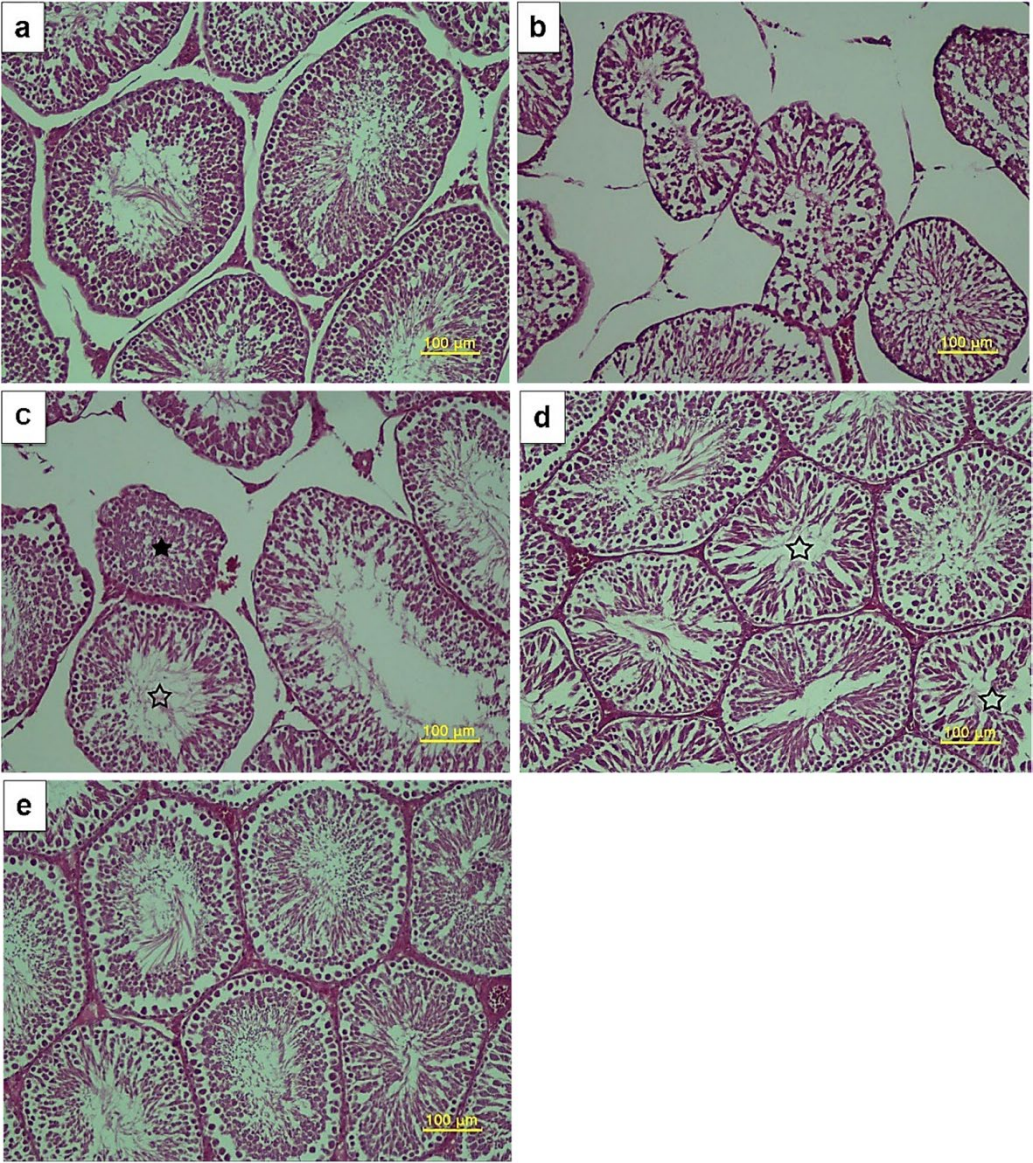
Representative photomicrographs of testis in Control (**a**), Diabetic (**b**), Diabetic + G (**c**), Diabetic + C (**d**), and Diabetic + GC (**e**) groups. In c, some tubules show a relatively normal structure (hollow star) and some tubules show a degenerative structure (filled star). In d, a number of tubules do not have a complete spermatogenesis process (hollow stars) (H & E, Scale bars: 100µm). For seminiferous epithelial height (I), seminiferous tubular diameter (II), meiotic index (III), spermatogenesis (IV) and Johnson’s score (V), values are mean ± SEM. *** *p* < 0.001 against Control group. ** *p* < 0.01 against Control group. * *p* < 0.05 against Control group. ### *p* < 0.001 against Diabetic group. ## *p* < 0.01 against Diabetic group. # *p* < 0.05 against Diabetic group. $$ *p* < 0.01 against Diabetic + G group. $ *p* < 0.05 against Diabetic + G group. Diabetic + G: Diabetic rats treated with garlic at a dose of 0.5 ml/100g BW. Diabetic + C: Diabetic rats treated with *C. colocynthis* at a dose of 5 mg/kg BW. Diabetic + GC: Diabetic rats treated with garlic and *C. colocynthis* at a dose of 0.5 ml/100g BW and 5 mg/kg BW respectively

In the Control group, most of the seminiferous tubules were round or oval with a flat base membrane. The epithelium of the seminiferous tubules was completely cell-filled and contained all germ cell lines. Also, inside of most tubules was full of spermatozoa (Fig. 5a). Microscopic examination of sections of the Diabetic group revealed that some of the tubules were no longer round or oval in shape and had shrinkage or collapse. Most of the germ cells were destroyed and the number of tubules that did not show spermatozoa inside their ducts increased compared to the control group (Fig. 5b). Also, in the Diabetic + G group, with the exception of a few seminiferous tubules, the rest were full of cells and spermatozoa were even seen inside the duct (Fig. 5c). In the Diabetic + C group, less pathological lesions were seen than in the Diabetic group, but it was still far from the normal structure of the testis (Fig. 5d). The Diabetic + GC group had the best protective effect among the treatment groups. Most of the seminiferous tubules were round or oval and full of cells, and all germ cell lines were located regularly in the epithelium, well indicating the natural process of spermatogenesis in these tubules (Fig. 5e). The height of seminiferous epithelial in Diabetic (*p* < 0.01) and Diabetic + C (*p* < 0.05) groups showed a significant decrease compared to the Control group. All three treatment groups slightly increased the height of the seminiferous epithelial relative to the Diabetic group (Fig. 5I). The diameter of seminiferous tubules in Diabetic (*p* < 0.001), Diabetic + G (*p* < 0.05) and Diabetic + C *p* < 0.01) groups was notably decreased compared to the Control group. But the Diabetic + G and Diabetic + C groups (*p* < 0.05) and the Diabetic + GC group (*p* < 0.01) showed a notable increase compared to the Diabetic group (Fig. 5II). Meiotic index decreased significantly in Diabetic and Diabetic + C (*p* < 0.01) groups compared to the Control group. However, the Meiotic index in the Diabetic + GC group increased significantly compared to the Diabetic (*p* < 0.01) and Diabetic + C (*p* < 0.05) groups (Fig. 5III). In addition, the percentage of spermatogenesis in the Diabetic group (*p* < 0.001) and Diabetic + G and Diabetic + C groups (*p* < 0.01) relative to the control group showed a significant decrease. However, the percentage of spermatogenesis in all treatment groups increased significantly compared to the Diabetic group (*p* < 0.001). It showed a significant increase in the Diabetic + GC group (*p* < 0.05) compared to the Diabetic + G group (Fig. 5IV). Johnson's score was significantly lower in the Diabetic, Diabetic + G and Diabetic + C groups (*p* < 0.001) and the Diabetic + GC group (*p* < 0.01) compared to the Control group. Nevertheless, Johnson's score in all three treatment groups illustrated a significant increase relative to the Diabetic group (*p* < 0.001). Also, the Diabetic + GC group had a notable increase compared to the Diabetic + G group (*p* < 0.01) (Fig. 5V).

### Immunohistochemical evaluation in the testis

**Fig. 6:**
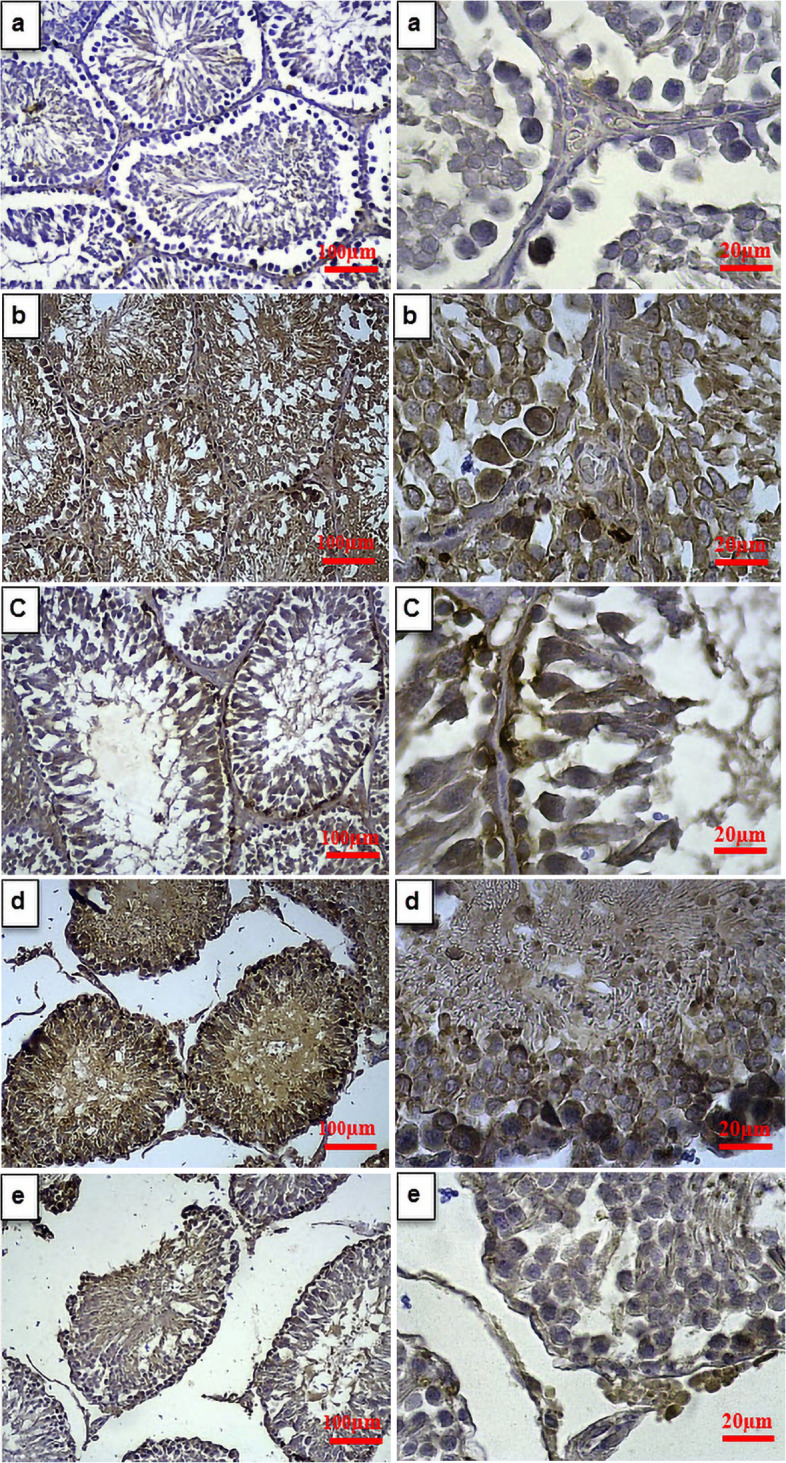
Caspase-3 immunoexpression in the testis of Control (**a**), Diabetic (**b**), Diabetic + G (**c**), Diabetic + C (**d**), and Diabetic + GC (**e**) groups (Scale bars: 100µm and 20µm; For left and right photos, respectively). For expression of caspase-3 (I), values are mean ± SEM (*n* = 3). *** *p* < 0.001 against Control group. ### *p* < 0.001 against Diabetic group. $$$ *p* < 0.001 against Diabetic + G group. $$ *p* < 0.01 against Diabetic + G group. &&& *p* < 0.001 versus Diabetic + C group. Diabetic + G: Diabetic rats treated with garlic at a dose of 0.5 ml/100g BW. Diabetic + C: Diabetic rats treated with *C. colocynthis* at a dose of 5 mg/kg BW. Diabetic + GC: Diabetic rats treated with garlic and *C. colocynthis* at a dose of 0.5 ml/100g BW and 5 mg/kg BW respectively

Figure 6 a, b, c, d & e shows the effect of garlic and *C. colocynthis* separately and in combination on caspase-3 immunoexpression. The expression of caspase-3 in all groups showed a significant increase compared to the Control group (*p* < 0.001). However, its expression in Diabetic + G and Diabetic + GC groups was significantly reduced relative to the Diabetic group (*p* < 0.001). Caspase-3 expression was also significantly reduced in Diabetic + GC group compared to Diabetic + G and Diabetic + C groups (*p* < 0.001). Also, its expression was significantly increased in the Diabetic + C group compared to the Diabetic + G group (*p* < 0.01) (Fig. 6I).

## Discussion

DM is a pathological condition that has adverse impacts on several systems including the reproductive system [34] and Garlic and *C. colocynthis* have been widely used to treat these conditions [22, 35, 36]. This is the first report to prove the synergistic therapeutic effects of garlic and *C. colocynthis* in combination on DM-induced male reproductive injury. The protective effects may be related to hormone regulation, anti-oxidant and anti-apoptotic effects of garlic and *C. colocynthis*, possibly by suppressing the activation of the AGEs/RAGE/Nox-4 pathway.

In this study, blood glucose in diabetic rats increased remarkably compared with control rats, which is consistent with other studies [36, 37]. STZ causes selective loss of insulin-secreting pancreatic β cells through reactive oxygen species-dependent oxidative damage [38]. High blood glucose causes a reduction in structural proteins, muscle wasting and eventually a severe weight loss in diabetic rats [39]. Consistent with other report [36], in our study, diabetic rats showed weight loss. We found that testis weight in diabetic rats was significantly reduced compared to control rats, which is consistent with previous studies [36, 40]. Individual administration of garlic and *C. colocynthis* slightly reduced FBG in diabetic rats but not significantly. Previous studies have shown a significant effect of these two plants on reducing FBG when used separately. This difference can be due to the difference in dosage [28, 41]. Using the combination of these two plants has been able to significantly reduce FBG. Garlic extract leads to insulin secretion, suppression of glucagon secretion, increased glucose utilization and energy metabolism in skeletal muscle by inhibiting the enzyme dipeptidyl peptidase-4, thereby lowering blood glucose [42]. *C. colocynthis* fruit reduces blood glucose by increasing insulin-induced GLUT4 translocation and glucose uptake into adipocytes by increasing phosphorylation of protein kinase B [35]. Moreover, the combination of garlic and *C. colocynthis* may cause a significant decrease in blood sugar by using these pathways. Therefore, we found that combination form could increase body and testis weight in diabetic rat, whereas individual consumption was slightly effective.

In diabetes, testis steroidogenesis and serum levels of FSH and LH are suppressed. Testosterone from Leydig cells, is essential for sperm production and improved secondary sexual characteristics [43]. We saw diabetes significantly reduced serum LH and testosterone levels, consistent with previous studies [36, 44]. We also observed that LH and testosterone levels increased slightly in the garlic and *C. colocynthis*-treated groups, but their levels in the combination-treated group showed a significant increase. Catechin has increased testosterone levels in rats by inhibiting aromatase activity [45]. Quercetin improves testosterone production in rats exposed to cadmium chloride or the herbicide atrazine by restoring the enzymatic activity of 3β-HSD and 17β-HSD [46, 47]. It also improves testicular function by increasing mRNA expression level of beta subunit of LH gene in the pituitary and LH receptor gene in the testis [48]. Also, diallyl sulfide increases LH levels and increases the activity of 17β-HSD and 3β-HSD enzymes [49]. Therefore, there is a possibility that the positive effect of the combination of garlic and *C. colocynthis* can be achieved by the above mechanisms.

Decreased sperm count and histopathological analysis of diabetic rats confirm impairment of spermatogenesis, as reported in other studies [36, 40, 50]. All three treatment groups showed increased sperm count, Johnson’s score, spermatogenesis percentage, and meiotic index, but this increase was more pronounced in the combination group.

Chronic hyperglycemia leads to the formation of AGEs, a heterogeneous group of modified proteins resulting from non-enzymatic reaction between the glucose carbonyl group and its derivatives with the free amino group of proteins. The present study, like previous studies, showed that the level of AGEs in diabetic rats increased significantly compared to control rats [51]. AGEs causes excessive ROS production, leading to oxidative damage [52]. What is worse, ROS can in turn promote the formation of AGEs [53]. Administration of garlic and *C. colocynthis* decreased AGEs and RAGE, with a significant effect in the combined group. Garlic showed significant levels of protection in protein structural stabilization against glycation [54]. Moreover, although the effect of *C. colocynthis* on AGEs has not been studied yet, but the mechanism of quercetin and catechin has been investigated. Quercetin prevents AGEs formation by chelating metal ions, trapping methylglyoxal, and trapping ROS [55]. Catechin inhibits AGE formation by its antioxidant capacity, 1,1-diphenyl-2-picrylhydrazyl scavenging ability, trapping of methylglyoxal, protecting of protein structure, inhibiting activities of α-amylase, α-glucosidase and β-glucosidase [53].

ROS overproduction causes lipid peroxidation and mitochondrial damage in germ and Leydig cells, leading to impairment in spermatogenesis and steroidogenesis [56]. We observed increased significant levels of MDA and H_2_O_2_ in the testis of diabetic group, which is consistent with the study of Adedara et al. [36].

The antioxidant system plays an essential role in protecting cells against oxidative damage by ROS [36]. This system contains low molecular weight antioxidants such as glutathione and enzymatic antioxidants such as CAT and GPx [57]. The decreased CAT activity observed in diabetic rats simply indicates inhibition of its antioxidant function. In addition, a significant reduction in the activity of the GSH-dependent enzyme, GPx, in diabetic rats may indicate enzyme inhibition due to a decrease in substrate (i.e. GSH), as shown in previous study [36]. GSH-mediated GPx action results in elevation of GSSG [58]. GSSG level of the diabetic group was significantly increased compared to the control group, also observed on the brain and spinal cord [59]. Also, the level of FRAP, which is an indicator of antioxidant capacity, decreased in diabetic rats. In agreement with this study, Ghanbari et al. reported that the level of FRAP was decreased in the testis, pancreas and liver of the diabetic group [60, 61]. We demonstrated garlic and *C. colocynthis* improve oxidative stress by reducing MDA and GSSG, increasing CAT and GPx activity, and increasing GSH in diabetic rats, which were significant in the group treated with combination form of garlic and *C. colocynthis*. Thus, our results are in harmony with Lotfi et al., who stated that garlic reduces oxidative stress in the testis of diabetic rats [62]. Ostavan et al., also showed that *C. colocynthis* at a dose of 10 mg/kg BW for two weeks improves oxidative stress in the testes of diabetic rats [41]. Garlic effect is performed through its antioxidant potential [63]. Diallyl disulfide acts as H_2_S donors and inhibits oxidative stress by increasing the diminished concentration of GSH [64], and by increasing the local concentration of Nrf2 in the nucleus and thus regulating the gene expression of many antioxidant enzymes [65]. *C. colocynthis* has also been reported to have antioxidant activities [66]. The antioxidant effect of catechin is achieved through (I) direct mechanisms—elimination of ROS, chelation of metal ions and (II) indirect mechanisms—induction of antioxidant enzymes, inhibition of pro-oxidant enzymes, and production of phase II detoxification enzymes and antioxidant enzymes [67]. Antioxidant activity of quercetin is manifested mainly through its effect on GSH, enzymatic activity, signal transduction pathways, and ROS [68]. According to these explanations, the consumption of garlic and *C. colocynthis* together can lead to the synergy of their antioxidant effect.

AGEs exert their effects through receptors for advanced glycation end products (RAGE) [69] in which their interaction initiates multiple signaling pathways, such as Nox pathway [70]. The Nox family are proteins that transfer electrons across biological membranes. The electron receptor is oxygen and the product is superoxide, a ROS [71]. Nox-4 converts approximately 90% of the electron flux into H_2_O_2_ and 10% of it into O_2_^•−^. Expression of Nox-4 and RAGE increased significantly in diabetic group as confirmed by another study [72]. By administering garlic and *C. colocynthis*, their expression decreased, but the combination form was more effective. Also, Abdel-Mageid et al. showed garlic extract caused down-regulation of RAGE gene expression in the heart [73]. The effect of *C. colocynthis* on RAGE has not been studied yet, but catechin prevents its expression in the kidney of diabetic rats [74]. Cao et al., reported that diallyl sulfide reduces the expression and activity of Nox in macrophages to some extent [75]. Tian et al., reported that quercetin decreased Nox expression in endothelial of diabetic rats [76].

A target of RAGE signaling is NF-кB [77] which plays a major role in the transduction of inflammatory and pro-apoptotic signals, and RAGE-dependent activation of NF-κB leads to upregulation of RAGE itself [78]. In diabetes, signal for testis apoptosis rises. Caspase 3 is one of the main factors of apoptosis and its activation indicates irreversible cell apoptosis [79]. We showed that NF-кB expression and caspase-3 immunoexpression in diabetic rats had a significant increase compared to the control group, as seen in another study [50]. Therefore, activation of the AGEs/RAGE/Nox-4 signaling pathway is an important mechanism in causing DM-induced reproductive damage. Our findings also showed that garlic and *C. colocynthis* reduced NF-κB expression in all three treated groups, which was more effective in the combination group. In addition, the anti-apoptotic efficacy of these plants was evaluated and we found that immunoexpression of caspase-3 was significantly reduced in the diabetic group treated with garlic and also in the combination group. One study showed that diallyl sulfide reduces NF-kB expression in bleomycin-induced pulmonary fibrosis [80]. Another study found that diallyl disulfide decreased caspase-3 expression in the hearts of diabetic rats. As mentioned, diallyl disulfide acts as H_2_S donor that sulfhydrates post translationally the Cys^38^ residue of NF-κB, which in turn is activated to bind to its activator ribosomal protein S3, thus transcribing several anti-apoptotic genes [63]. Kumar et al., also showed that quercetin reduced the expression of NF-κB and caspase-3 in the retinas of diabetic rats [81].

## Conclusion

In summary, our findings indicate that the combination of garlic and *C. colocynthi*s improves DM-induced reproductive injury than when used separately. This improvement is achieved through reduction of histological changes, increase of sex hormone level, and decrease of apoptosis and inflammation due to oxidative stress. The AGEs/RAGE/Nox-4 signaling pathway is involved in this injury. Further research is needed to identify other possible pathways, such as AGEs/RAGE/p38mapk, responsible for the protective effects of garlic and *C. colocynthi*s combination on male reproductive injury caused by DM. Moreover, in this study we showed with lower doses of garlic and *C. colocynthi*s, we could achieve the protective effects to lessen the prolonged consumption-induced side effects.

In addition, further studies are suggested on other medicinal plants to find a combination that have the higher efficacy with less side effects.

## Data Availability

Data are available from the corresponding author upon request. Email: agol@uk.ac.ir..

## Abbreviations

AGEs: Advanced Glycation End products
RAGE: Advanced Glycation End products
NOX-4: NADPH oxidase-4
ROS: Reactive Oxygen Species
DAS: Diallyl sulfide. DADS: Diallyl disulfide. DATS: Diallyl trisulfide. SAC: S-Allyl Cysteine. FBG: Fasting Blood Glucose
ELISA: Enzyme-Linked Immunosorbent Assay
PBS: Phosphate-Buffered Saline
GAPDH: Glyceraldehyde-3-phosphate dehydrogenase
NF-кB: Nuclear Factor kappa B
MDA: Malondialdehyde
CAT: Catalase
GPx: Glutathione Peroxidase
GSH: Glutathione
GLUT4: Glucose Transporter Type 4
FSH: Follicle-Stimulating Hormone
LH: Luteinizing Hormone
β-HSD: β-Hydroxysteroid dehydrogenase
GSSG: Glutathione disulfide
